# Pulmonary Metastasectomy for Adrenocortical Carcinoma—Not If, but When †

**DOI:** 10.3390/cancers16040702

**Published:** 2024-02-07

**Authors:** Shamus R. Carr, Frank Villa Hernandez, Diana Grace Varghese, Hyoyoung Choo-Wosoba, Seth M. Steinberg, Martha E. Teke, Jaydira Del Rivero, David S. Schrump, Chuong D. Hoang

**Affiliations:** 1 Thoracic Surgery Branch, Center for Cancer Research, National Cancer Institute, National Institutes of Health, Bethesda, MD 20892, USA; schrumpd@mail.nih.gov; 2 Surgical Oncology Program, Center for Cancer Research, National Cancer Institute, National Institutes of Health, Bethesda, MD 20892, USA; frank.villahernandez@nih.gov (F.V.H.); martha.teke@utsouthwestern.edu (M.E.T.); 3 Developmental Therapeutics Branch, National Cancer Institute, National Institutes of Health, Bethesda, MD 20892, USA; diana.varghese@nih.gov (D.G.V.);; 4 Biostatistics and Data Management Section, Center for Cancer Research, National Cancer Institute, National Institutes of Health, Bethesda, MD 20892, USA

**Keywords:** adrenocortical cancer, pulmonary metastasectomy, survival, observational case series

## Abstract

**Simple Summary:**

Adrenocortical carcinoma is a rare tumor with a very high propensity to metastasize to the lungs. There are limited systemic options once it does metastasize. Pulmonary metastasectomy has been utilized in these patients as part of a treatment paradigm. However, there is little information available to help guide patient selection for pulmonary metastasectomy. Herein, our experience treating adrenocortical patients with only lung metastases was reviewed to try and identify associations between different variables (e.g., number of metastases, time to resection) and outcomes. The results demonstrated that, unlike other malignancies, the total number of nodules should not preclude patients who have adequate pulmonary reserve. Additionally, the time from original surgery to the development of lung metastases may have prognostic implications. Future work should focus on identifying genetic markers associated with outcomes that can be used independently or in combination with other clinical variables.

**Abstract:**

Background: Adrenocortical carcinoma (ACC) commonly metastasizes to the lungs, and pulmonary metastasectomy (PM) is utilized due to limited systemic options. Methods: All ACC patients with initially only lung metastases (LM) from a single institution constituted this observational case series. Kaplan-Meier and Cox proportional hazard analyses evaluated the association with potential prognostic factors and outcomes. Overall survival (OS) was calculated from the date of the PM or, in those patients who did not undergo surgery, from the development of LM. Results: A total of 75 ACC patients over a 45-year period met the criteria; 52 underwent PM, and 23 did not. The patients undergoing PM had a median OS of 3.1 years (95% CI: 2.4, 4.7 years) with the 5- and 10-year OS being 35.5% and 32.8%, respectively. The total resected LM did not impact the OS nor the DFS. The patients who developed LM after 11 months from the initial ACC resection had an improved OS (4.2 years; 95% CI: 3.2, NR; *p* = 0.0096) compared to those developing metastases earlier (2.4 years; 95% CI: 1.6, 2.8). Patients who underwent PM within 11 months of adrenalectomy demonstrated a reduced OS (2.2 years; 95% CI: 1.0, 2.7) compared to those after 11 months (3.6 years, 95% CI: 2.6, NR; *p* = 0.0045). PM may provide benefit to those patients with LM at presentation (HR: 0.5; *p* = 0.2827), with the time to first PM as a time-varying covariate. Conclusions: PM appears to have a role in ACC patients. The number of nodules should not be an exclusion factor. Patients developing LM within a year of primary tumor resection may benefit from waiting before further surgeries, which may provide additional insight into who may benefit from PM.

## 1. Introduction

Despite initial questions about the utility of pulmonary metastasectomy (PM) as part of the treatment paradigm of lung metastases [1], this treatment option has been routinely recommended for decades [2,3,4,5]. Recently, the role of PM, specifically in colorectal cancer (CRC), has again been questioned [6,7]. However, in the treatment of CRC there exist systemic therapies that appear to rival PM in terms of overall survival (OS). While many other cancers develop lung metastases, extrapolating treatment strategies across tumor types should be avoided.

Adrenocortical carcinoma (ACC) is a rare disease where a large proportion of patients will develop recurrence or metastatic disease, with the lungs being the most common metastatic site (40–80%) [8,9]. This is compounded by few efficacious systemic therapy options for metastatic disease [10,11,12,13]. Some early studies suggested that PM could be considered an effective treatment option for patients with isolated lung metastases from ACC [14]. Further single-arm retrospective studies supported this claim, but none provide any insight into variables that affect outcomes when considering PM [15,16,17]. Repeat PM in ACC cases is lauded to benefit OS, but there are few studies with very few patients [18,19,20,21]. Thus, whether a true benefit exists remains unclear.

In 2011, our group published a review of twenty-six ACC patients who only had lung metastases and underwent PM [16]. At the time, it was one of the most comprehensive reviews of PM for ACC. We re-examined these patients, along with all new patients through 2022 to determine if our prior conclusions remained valid and if further insights could be gained regarding the management of these patients.

## 2. Methods

### 2.1. Data Collection

An observational case series review identified all patients diagnosed with ACC at the National Cancer Institute, Bethesda, Maryland, between 1977 and 2022 who had either (1) only lung metastases at the time of ACC diagnosis or (2) developed lung metastases as the initial site of recurrence. Patients whose first site of metastases was outside the lung were excluded. Medical records were reviewed and analyzed for patient demographics (Table 1), date of diagnosis and primary ACC resection, date of initial identification of lung metastases, date of PM, number of nodules resected, functional status of primary ACC tumor, date of recurrence, date(s) of re-recurrence or re-operative surgery, complications and length of stay after re-operative surgery, neoadjuvant and adjuvant chemotherapy for either primary ACC or lung metastases, overall survival (OS), disease-free survival (DFS), and progression-free survival (PFS). All the patients were enrolled in various Institutional Review Board-approved investigational protocols from either the National Institutes of Diabetes and Digestive and Kidney Disease (NIDDK) or the National Cancer Institute (NCI). Written informed consent was obtained prior to any interventions, as per protocol for participation in these clinical studies. The sharing of deidentified data can be considered after contact with the corresponding author.

### 2.2. Statistical Analysis

The OS for those patients who underwent PM was defined as the time until death or until the last follow-up after the first PM. The DFS and PFS were similarly defined as the length of time with no evidence of metastases after the first resection without a subsequent resection, or the length of time with evidence of metastases after the first resection without a subsequent resection, respectively. For the patients who underwent planned staged bilateral PM, the date of the second operation was used as the starting point for these calculations. In those who did not undergo PM, the starting point for OS was the initial identification of lung metastases.

Among all the patients, our analyses were based on quartiles of time between the initial ACC diagnosis and the initial lung metastases, which were defined as follows: Q1 ≤ 4.5 days; Q2 > 4.5 days and ≤326 days; Q3 > 326 days and ≤940 days; and Q4 > 940 days. Some analyses included only patients who underwent PM and were categorized into four equal-sized groups by quartiles of days between ACC diagnosis and the first pulmonary metastasis resection. These quartiles were defined as follows: Q1 ≤ 327.5 days; Q2 > 327.5 days and ≤947.5 days; Q3 > 947.5 days and ≤1875.5 days; and Q4 > 1875.5 days. Also, among those who underwent PM, an analysis of the total number of metastases resected was carried out, evaluated in four groups determined to make the sample sizes for each group as equal as possible. The following groups were defined: Q1 = 1; Q2 = 2 or 3; Q3 ≥ 4 and ≤10; and Q4 ≥ 11.

Kaplan-Meier (K-M) plots with medians and numbers at risk were provided. Survival probabilities with 95% confidence intervals (CIs) were provided at specific time points of corresponding outcomes as well. The *p*-values corresponding to K–M plots were exact, based on 100,000 random permutations.

In our analyses of the effect of a first PM following the diagnosis of lung metastases among all the patients, Cox proportional hazards modeling was used to estimate the hazard ratio (HR) of this factor, which was treated as a time-varying covariate, and determine the corresponding 95% CI for the HR. A similar analysis was carried out to ascertain the impact of a second metastatic resection among those who had an initial pulmonary resection.

It should be noted that all the statistical results for the analyses should be interpreted as exploratory, including interpreting *p*-values in this context. In analyses where the quartiles were combined after examining the initial results, the *p*-value reported was adjusted by multiplying by three to account for the implicit multiple comparisons resulting from reducing the categories from four to two groups (e.g., obtaining 0.0045 = 0.0015 (raw *p*-value) × 3). A statistical analysis was performed using SAS version 9.4 for Windows (Cary, NC, USA) and R version 4.1.1.

## 3. Results

A total of 80 patients were identified between 1977 and 2022. Fifty-two patients underwent at least one PM as part of their treatment. The remaining 28 patients did not undergo PM, 5 of whom were excluded due to a lack of information or failure to meet the inclusion criteria. The 23 patients who did not undergo PM did so for two main reasons: patient choice or poor pulmonary status. This resulted in a total of seventy-five patients, of which all but four received standard-of-care chemotherapy. Of these patients, 20 were part of the analysis from our institution published in 2011 [16]. The majority of operations consisted in open thoracotomy with a Perelman technique to preserve lung parenchyma [22]. However, VATSs (video-assisted thoracic surgery) with stapled wedge resections were also employed at the discretion of the surgeon (Figure S1).

### 3.1. All Patients

**Figure 1:**
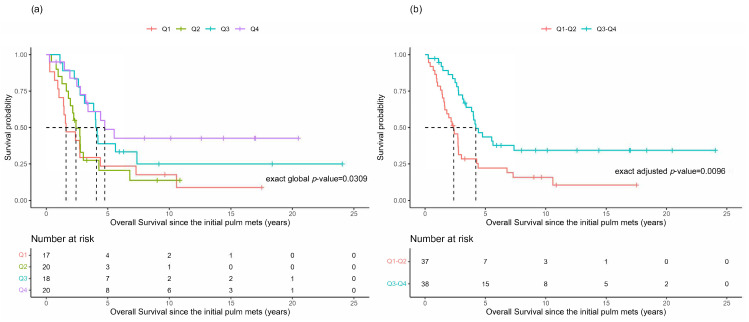
Overall survival based upon the time to the initial identification of lung metastases. Kaplan-Meier plots for the overall survival (OS) of patients based upon the time between the primary ACC diagnosis and the initial identification of lung metastases (${Time}_{diag-initPM}$) grouped by quartiles (**a**) or re-categorized as Q1–Q2 versus Q3–Q4 (**b**).

Overall survival was evaluated in all 75 patients using the time interval between the primary ACC diagnosis and the initial development of lung metastases as a time-varying covariate. There was a potential difference in the OS between the four quartiles: Q1 ≤ 4.5 days; Q2 > 4.5 days and ≤326 days; Q3 > 326 days and ≤940 days; and Q4 > 940 days (Figure 1a, *p* = 0.0309). It was noted that the OS probabilities in Q1 and Q2 seemed similar, with respect to the median OS, while Q3 and Q4 appeared to have similar but higher median OS times (Table 2). The re-categorization into two groups of Q1–Q2 and Q3–Q4 is shown in the K–M plots (Figure 1b, *p* = 0.0096). It is noticeable that the overall survival distributions are different between these re-categorized two groups, with corresponding small *p*-values (*p* = 0.0096).

Additionally, a Cox proportional hazard analysis was performed to assess if there was any statistical difference in the hazard rates for the OS associated with the time-varying covariate of the first PM within each of the quartiles as defined by time from ACC diagnosis to the appearance of lung metastases. No statistically meaningful associations were found; there was no noticeable difference in the HR based on the patients with and without a first metastasis resection for any subgroup (Table 3). It could be noted that, in the 17 patients included in Q1, there was a potential effect of the benefit of an initial metastasis resection among those having initial pulmonary metastases at the same time as their primary ACC diagnosis (HR = 0.50; *p* = 0.28). This observation is based on a limited number of patients and would need to be interpreted cautiously.

### 3.2. Patients Undergoing PM

Analyses restricted to the 52 patients undergoing PM demonstrated a median OS of 3.1 years (95% CI: 2.4, 4.7 years) from the time of PM (Table 4). The survival probability decreased quite rapidly and then stabilized, resulting in OS at 5 and 10 years after PM of 35.5% and 32.8%, respectively. The median DFS and PFS after PM were 1.5 years and 3 months, respectively.

**Figure 2:**
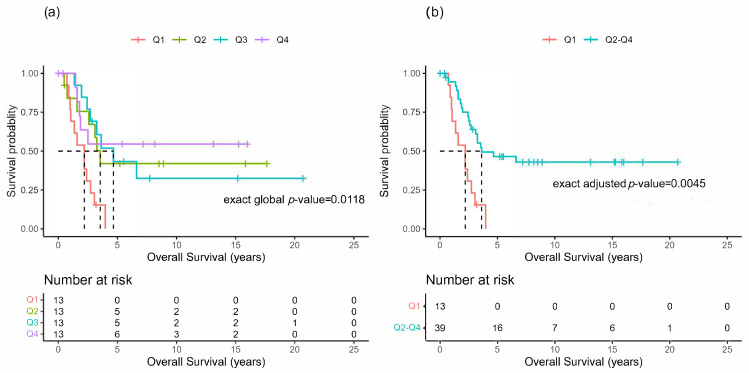
Overall survival after pulmonary metastasectomy. Kaplan-Meier plots for the overall survival (OS) of patients undergoing pulmonary metastasectomy grouped by quartiles of the time interval between the primary resection of ACC and the initial PM resection (${Time}_{resc-initPM}$) (**a**) or re-categorized as Q1 versus Q2–Q4 (**b**).

When grouped in quartiles according to the time between the primary ACC resection and the first pulmonary metastasis resection, the survival distributions of the OS appeared to be potentially different among the four groups (Figure 2a), with Q1 seemingly having a much shorter OS, as there were no survivors after 5 years, compared with the other groups. Thus, re-categorizing into two groups, Q1 versus Q2–Q4 (Figure 2b), demonstrated a significant survival difference (*p* = 0.0045). The median OS for Q1 was 2.2 years (95% CI: 1.0, 2.7), and for Q2–Q4 it was 3.6 years (95% CI: 2.6, NR).

**Figure 3:**
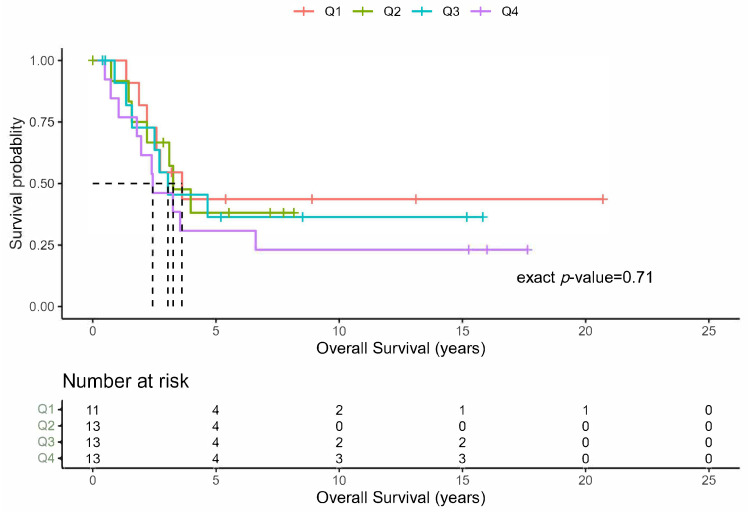
Overall survival by total number of resected lung metastases. Kaplan-Meier plots of the overall survival for the total number of initial lung metastases resected grouped by quartiles ($N_{initPM}$).

The median number of PM resected per patient was eight (range 1–75). The grouping of the patients into four groups of approximately equal sample sizes revealed that there was no difference in the OS (Figure 3; *p* = 0.71), DFS (*p* = 0.18), or PFS (*p* = 0.19). Lastly, the patients undergoing PM were analyzed based upon the functional status of the primary tumor. These results do not demonstrate any recognizable differences in the distributions of the OS, DFI, or PFS, reflected by corresponding large *p*-values between those with and without functional ACC tumors.

Cox proportional hazard model analysis was used to test whether the OS differed according to whether the patients did or did not experience an additional metastasis resection. This factor was considered as a time-varying covariate in the model since subsequent resections may take place at various times after pulmonary metastasis diagnosis. The test resulted in a hazard ratio of about 0.66, with a corresponding *p*-value of 0.27. This implied that the hazard rate in the patients with a subsequent metastasis resection was about 34% lower on average than in the patients with only a primary PM. However, this magnitude of the difference in hazard rates might not indicate much of an effect, with a relatively large *p*-value (>0.2).

## 4. Comment

It is not uncommon for ACC to initially present with distant metastatic disease to the lungs. In addition, 40–80% of patients with ACC who do not have stage IV disease at presentation will develop pulmonary metastases [8,9]. Compounding the situation are limited efficacious options for systemic treatment [13,23]. Systemic therapy consists of either mitotane alone or in combination with other cytotoxic therapies [11,13,24,25]. Nevertheless, response rates are poor. These factors combined have propelled the consideration of surgical resection (PM and repeat PM) as part of the treatment paradigm for ACC when pulmonary metastatic disease is identified, despite limited evidence in the literature [14,16,17,18,24,26].

Understanding the actual survival and recurrence rates of patients with metastatic ACC is complex. Nearly all publications include heterogenous grouping of patients with the two most common sites of metastases: lung and liver. In these studies, the reported median survival after undergoing resection of metastatic disease is between 14 and 30 months [16,17,18,26]. A disease-free interval (DFI) of less than 12 months is associated with poor survival [17]. Another factor that negatively affects outcome includes the development of abdominal metastases [9]. Therefore, in order to better understand and more optimally guide the management of ACC patients with lung metastases, it is important to look solely at a homogenous population of patients with metastatic or recurrent disease confined to the lungs in the absence of extrathoracic recurrence. Findings gleaned from this selective cohort may enhance our understanding of what treatment options should be offered to patients.

We analyzed our 45-year single-institution experience to identify factors for improved patient selection and outcomes (OS). Our 2011 data suggested potential benefits of PM in well-chosen patients [16]. This was based on 26 patients, including 6 with concurrent extrapulmonary disease during PM. Neither adjuvant nor neoadjuvant chemotherapy were associated with an improved OS. Limited by the sample size and a quarter of patients having extrathoracic metastases, more definitive conclusions could not be drawn. Yet, the 5-year actuarial survival of 41% was consistent with the 35.5% that we now report.

Disease-free interval has been shown in many histologies as an influential factor associated with outcomes [5,27]; ACC is no different. However, non-ACC histologies with improved outcomes are usually associated with a DFI greater than 2 years. Our results (Figure 2) and others demonstrate that a DFI of at least 11 months in ACC is associated with an improved OS [17]. However, this study included patients with local and distant recurrence. Our results imply that those patients with only lung metastases who undergo an initial PM within 11 months of their primary ACC tumor resection are expected to have about a 1.4 year shorter median OS than those who undergo PM after 11 months. This does not mean that delaying PM improves the OS but may indicate that a delay before PM provides improved identification of either lung metastases or local recurrence. This period of observation indirectly acts as a surrogate evaluation of an individual tumor biology, allowing improved selection of patients in whom PM will be of benefit. In other words, are the initially seen lung metastases the complete picture or just the “tip of the iceberg”?

Yet, careful consideration of patients who have metastatic disease at the initial presentation of their primary tumor compared to those who develop lung metastases within 11 months does show a difference. In fact, patients with lung metastases at the time of initial presentation of their ACC tumor who undergo PM have a 50% lower hazard ratio compared with the patients who did not (Table 3). However, the *p*-value is relatively large, and there is a relatively large standard error for the HR estimate of 0.5. Further scrutiny of the results in Table 3 could be interpreted as not having a survival advantage to patients who undergo PM, regardless of the timing. However, the actual OS (Table 4) demonstrates a 5-year survival of 35.5% where it plateaus and remains essentially unchanged for up to 10 years (32.8%) in those undergoing PM in our study of patients accrued over 45 years. Yet, due to the small number of patients in various groups who did not undergo any resection due to an implicit selection bias for surgery, definitive conclusions with respect to recommendations about who should and should not undergo PM are still lacking and require further study to include a genetic evaluation of both the primary tumor and lung metastases.

When considering PM, it is generally accepted that the total number of lung lesions is prognostic and indirectly associated with the outcomes, but this has not been explored previously in ACC. Most histologies find that a number greater than four pulmonary lesions tends to be associated with poor survival and that PM should not be recommended in these patients [5,28]. One possible exception may be in patients with primary sarcoma with 10 or more metastases among who there was an OS at 5 and 10 years of 26% and 17%, respectively [3]. Thus, ACC seems to be similar to sarcoma lung metastases in this regard. While the median number of metastatic lung nodules resected in our study was eight, the range was from one to seventy-five. When separating the patients into equal quartiles, there was no meaningful difference in the OS for patients with a single metastatic lung lesion or those with more than eleven lung metastases (Figure 3). Therefore, it appears that, if a patient has an adequate pulmonary reserve, the total number of lung lesions should not preclude PM.

Although this analysis provides an updated comprehensive evaluation of the role of PM in one of the largest single institutional experiences of patients with only pulmonary metastases from ACC, our analysis suffers from some key limitations. The retrospective nature of this study over 45 years and the limited sample size within groups continue to affect our findings. However, the majority of the patients in our study were treated in the past 15 years (Supplemental Figure S1), thus providing a contemporary perspective. While technology and surgical approaches have advanced during the time of this study, these likely had a limited impact on the outcomes. An important factor associated with long-term survival is the ability to resect all disease, whether by VATS or open-chest techniques. The other important factor is the inherent biology of the individual tumor, which is under investigation. Additionally, referral bias to our institution and implicit selection bias for surgery may also limit some of our conclusions.

## 5. Conclusions

ACC with lung metastases poses challenges due to limited systemic options and the absence of a genetic signature for guiding PM recommendations. It appears that there is a role for PM in ACC patients with isolated lung metastases based upon 5- and 10-year OS rates of 35.5% and 32.8%, respectively. The development of lung metastases after resection of the primary ACC tumor, especially in the first year, is associated with worse overall outcomes despite PM. A prudent strategy of careful surveillance may provide insight into individual tumor biology and help delineate those patients who would benefit from surgery. PM potentially extends a patient’s OS when lung metastases appear more than a year after the primary ACC resection. The total number of nodules should not sway patient selection. For stage IV cases with only lung metastases at diagnosis, the role of PM is uncertain due to insufficient data. Future work should explore genetic and sequencing analyses to enhance treatment strategies.

## Figures and Tables

**Table 1:** Patient demographics.

Variable	No. (%)
Feature:	
Sex, M/F	16/59
Age at primary adrenalectomy, non-PM group, median, years (range)	50 (27–59)
Age at primary adrenalectomy, PM group, median, years (range)	41 (9–68)
Age at first PM, median, years (range)	45 (9–72)
Number of lung metastases, median (range)	8 (1–75)
Pulmonary Metastasectomy:	
Yes	52 (69)
1–2 PMs	33 (63)
3–5 PMs	16 (31)
5–7 PMs	3 (6)
No	23 (31)
Hormonal Status at Presentation:	
Functional	41 (55)
Hypercortisolism	26 (63)
Hyperaldosteronism	8 (20)
Virilization	5 (12)
Feminization	2 (5)
Nonfunctional	31 (41)
Unknown	3 (4)
Systemic Therapy:	
Yes	71 (95)
No	4 (5)
Systemic Therapy PM Group:	
Induction Chemotherapy	5 (10)
Adjuvant Chemotherapy	46 (88)
Systemic Therapy Non-PM Group:	
Yes	23 (100)

**Table 2:** Overall survival (OS) from ACC diagnosis until development of lung metastases.

	Median (95% CI)						*p*-Value ^a^	
	Q1	Q2	Q3	Q4	Q1–Q2	Q3–Q4	Global ^b^	As Grouped ^c^
OS (years)	1.6 (0.9, 4.4)	2.4 (1.6, 3.0)	4.1 (2.8, 7.4)	4.8 (2.8, -)	2.4 (1.6, 2.8)	4.2 (3.2, -)	0.031	0.0096 (0.0032)

^a^ an exact log rank *p*-value is derived from 100,000 random permutations. ^b^ a global test-based *p*-value is based on a test for the null hypothesis of equal survival distributions among all four groups. ^c^ a Bonferroni-based adjusted *p*-value with raw *p*-value in parentheses. Quartiles defined as follows: Q1 ≤ 4.5 days; Q2 > 4.5 days and ≤326 days; Q3 > 326 days and ≤940 days; and Q4 > 940 days.

**Table 3:** Hazard ratios if pulmonary metastasectomy was performed based upon the time patients took to develop lung metastases.

Quartiles Subgroups	N ^a^	$N_{yes}$ ^b^	$N_{no}$ ^c^	HR ^d^	*p*-Value ^e^
Q1	17	7	10	0.50	0.2827
Q2	20	13	7	1.36	0.5588
Q3	18	16	2	1.33	0.7287
Q4	20	16	4	0.99	0.9838
Q1–Q2	37	20	17	0.90	0.7899
Q3–Q4	38	32	6	1.20	0.7212

Q1: ${Time}_{diag-initPM}$ ≤ 4.5 days; Q2: 4.5 days < ${Time}_{diag-initPM}$ ≤ 326 days; Q3: 326 days < ${Time}_{diag-initPM}$ ≤ 940.50 days; and Q4: ${Time}_{diag-initPM}$ > 940.50 days. ^a^ the number of patients used within the subgroup. ^b^ the number of patients undertaking their first metastasis resection at the end of the study. ^c^ the number of patients not undertaking their first metastasis resection at the end of the study the number of patients not undertaking the first metastasis resection at the end of the study. ^d^ estimated hazard ratio derived from a Cox proportional hazard model with the first metastasis resection indicator as a time-varying covariate within the subgroup. ^e^ *p*-value derived from a Cox proportional hazard model with the first metastasis resection indicator as a time-varying covariate within the subgroup.

**Table 4:** Survival analysis summary of only patients undergoing pulmonary metastasectomy.

Outcome	Median (95% CI)	Survival Probability (95% CI)	
OS		5 years	10 years
	3.1 years (2.4, 4.7)	35.5% (22.3%, 49.0%)	32.8% (19.8%, 46.4%)
DFS		1 year	2 years
	1.5 years (0.7, 2.2)	57.1% (33.8%, 74.9%)	33.3% (14.9%, 53.1%)
PFS		6 months	12 months
	3.0 months (1.9, 9.9)	39.7% (22.5%, 56.5%)	27.8% (12.9%, 44.9%)

## Data Availability

Data are contained within the article and supplementary materials.

## Supplementary Materials

The following supporting information can be downloaded at https://www.mdpi.com/article/10.3390/cancers16040702/s1, Figure S1: Pulmonary Metastasectomies by Year.

## Abbreviations

ACC	adrenocortical carcinoma
CI	confidence interval
CRC	colorectal carcinoma
DFI	disease-free interval
DFS	disease-free survival
HR	hazard ratio
K-M	Kaplan-Meier
LM	lung metastases
NCI	National Cancer Institute
NIDDK	National Institutes of Diabetes and Digestive and Kidney Disease
OS	overall survival
PFS	progression-free survival
PM	pulmonary metastasectomy
Q1	first quartile
Q2	second quartile
Q3	third quartile
Q4	fourth quartile
VATS	video-assisted thoracoscopic surgery
