# Supplementary figures and images for: Pulmonary Metastasectomy for Adrenocortical Carcinoma—Not If, but When
Source: Cancers (Basel). 2024 Feb 7;16(4):702. doi: 10.3390/cancers16040702 (PMC10886862; doi:10.3390/cancers16040702)

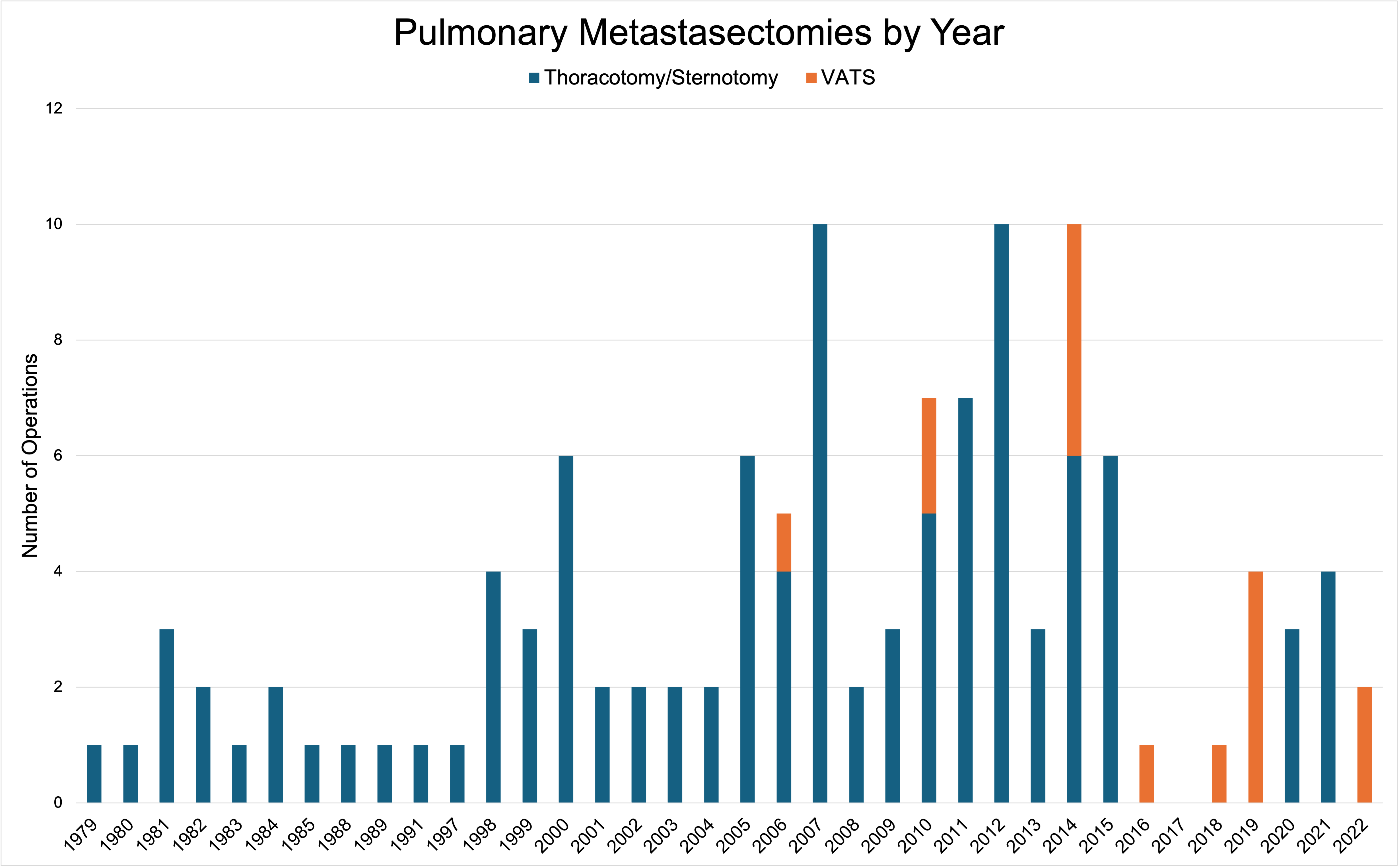

Supplement: Supplementary file 1 [file cancers-16-00702-s001.zip › Figure S1. Pulmonary Metastasectomies by Year.png]
